# Sprengel: Communication in Regard to One Hundred and Thirty-one Cases of Carcinoma of the Breast, Operated upon in Volkmann’s Clinic, during the Years 1874-78

**Published:** 1883-08

**Authors:** 


### Sprengel: Communication in Regard to One Hundred and Thirty-one Cases of Carcinoma of the Breast, Operated upon in Volkmann's Clinic During the Years 1874–78.

This is a condensation of a contribution to Langenbeck's *Archiv*, consisting mainly of statistics. Puerperal diseases have occurred in many of the cases. About thirty per cent. of those who had borne children, and afterward were afflicted with cancer, had suffered from mastitis. One is very likely to draw certain inferences from this fact. The site of the disease was most frequently upon the upper and outer side of the breast. Infiltration of the glands in the axilla usually followed the first appearance of the growth, after fourteen months, union with the skin after sixteen months, with the muscles after eighteen months. Ulceration occurred, upon an average, after twenty months. Metastases were observed, upon an average, two years after the local affection first made its appearance, and they were almost invariably accompanied by glandular infiltrations. He does not think that clinical facts support Török and Wittelshöfer in their statement that an extension of carcinoma along the direct course of the blood circulation is relatively common. The question of recurrence is all important. After the first operation, the average duration of time before the re-appearance of growth is eight months, after the second it is seven, after the third it is two months. When the breast alone is amputated, the disease often returns in the axillary glands, but if these glands have been removed, the recurrence is not usually in the axilla. Of the cases tabulated, twelve were radically cured, more than three years having elapsed since the operation was performed. It is all important that the operation be performed early in the history of the tumor. Out of twenty-nine of the tabulated cases in which there was no glandular infiltration, six were cured, while of one hundred and two cases in which the axillary glands were involved, only nine were cured.

Three of the fifteen cases recorded as cured, died of cancer, not of the breast, after more than three years of immunity from the disease, leaving only twelve, as already stated, which could be looked upon as radically cured.—*The American Journal of Obstetrics.*

